# Statistical analysis plan for management of hypertension and multiple risk factors to enhance cardiovascular health in Singapore: the SingHypertension pragmatic cluster randomized controlled trial

**DOI:** 10.1186/s13063-020-05016-4

**Published:** 2021-01-19

**Authors:** John C. Allen, Benjamin Halaand, Rupesh M. Shirore, Tazeen H. Jafar, for SingHypertension Study Group

**Affiliations:** 1 Centre for Quantitative Medicine, Duke-NUS Medical School, Level 6, Academia, 20 College Road, Singapore, Singapore; 2 Division of Biostatistics, Population Health Sciences, University of Utah, Salt Lake City, USA; 3 Program in Health Services & Systems Research, Duke-NUS Medical School, 8 College Road, Singapore, Singapore

**Keywords:** Blood pressure, Hypertension, Motivational counseling, Single-pill combination, Telephone follow-up, Cardiovascular, Statistical analysis plan

## Abstract

**Introduction:**

Cardiovascular disease (O'Lone E, Viecelli AK, Craig JC, Tong A, Sautenet B, Herrington WG, et al., Am J Kidney Dis 76(1):109–20, 2020) remains the leading cause of death in Singapore. Uncontrolled hypertension confers the highest attributable risk of CVD and remains a significant public health issue with sub-optimal blood pressure (BP) control rates. The aim of the trial is to evaluate the effectiveness and cost-effectiveness of a multicomponent intervention (MCI) versus usual care on lowering BP among adults with uncontrolled hypertension visiting primary care clinics in Singapore. This article describes the statistical analysis plan for the primary and secondary objectives related to intervention effectiveness.

**Methods:**

The study is a cluster randomized trial enrolling 1000 participants with uncontrolled hypertension aged ≥ 40 years from eight primary care clinics in Singapore. The unit of randomization is the clinic, with eight clusters (clinics) randomized in a 1:1 ratio to either MCI or usual care. All participants will be assessed at baseline, 12 months, and 24 months with measurements of systolic and diastolic BP, antihypertensive and statin medication use, medication adherence, physical activity level, anthropometric parameters, smoking status, and dietary habits.

The primary objective of this study is to assess the effectiveness of MCI versus usual care on mean SBP at the 2-year follow-up. The primary outcome is SBP at 24 months.

SBP at baseline, 12, and 24 months will be modeled at the subject level using a likelihood-based, linear mixed-effects model repeated measures (MMRM) analysis with treatment group and follow-up as fixed effects, random cluster (clinic) effects, Gaussian error distribution, and adjustment to degrees of freedom using the Satterthwaite approximation. Secondary outcomes will be analyzed using a similar modeling approach incorporating generalized techniques appropriate for the type of outcome.

**Discussion:**

The trial will allow us to determine whether the MCI has an impact on BP and cardiovascular risk factors over a 2-year follow-up period and inform recommendations for health planners in scaling up these strategies for the benefit of society at large. A pre-specified and pre-published statistical analysis plan mitigates reporting bias and data driven approaches.

**Trial registration:**

ClinicalTrials.gov NCT02972619. Registered on 23 November 2016.

## Introduction

### Background and rationale

Cardiovascular diseases [1] including heart attacks, stroke, and heart failure are the leading causes of disability and death in Singapore [2]. Uncontrolled high blood pressure (BP) confers the highest risk of CVD. About one in four adults suffers from hypertension in Singapore and 50% have uncontrolled BP [3]. Reducing BP and lipid levels decreases the risk of CVD, and well-structured programs of care delivery are effective in lowering BP and preventing CVD [4].

The SingHypertension study is a cluster-randomized controlled trial conducted in eight SingHealth polyclinics in Singapore that investigates whether the proposed, structured multicomponent intervention (MCI) primary care program is more effective in reducing BP than existing practices in Singapore polyclinics. The MCI program consists of (1) algorithm-driven antihypertensive treatment for all hypertensive individuals using a single pill combination (SPC) of anti-hypertensive agents and lipid-lowering medications for all high-risk hypertensive individuals, (2) motivational conversation by nurses for high-risk hypertensive individuals, (3) telephone-based follow-up of all hypertensive individuals by polyclinic nurses, and (4) discounts on SPC antihypertensive medication. The study is unblinded and participants and researchers at the clinics will be aware of the treatment assignment.

A detailed description of the trial protocol giving a general description of analysis approaches and methodology was published previously [5]. A more detailed description of the statistical analysis plan is provided in this article as it relates to the major objectives of the study and the assessment of intervention effectiveness based on clinical and patient-reported outcomes. The purpose of an a priori statistical analysis plan is to avert post hoc reporting bias and data-driven analysis.

The outpatient primary health care system of Singapore is serviced by 18 subsidized government polyclinics. As previously described [5], the trial was planned to be conducted in eight clinics under a single administrative network. The unit of randomization is the polyclinic with clinics randomized 1:1 to MCI or usual care, with the randomization generated using computer code such that 4 clinics are assigned to MCI and 4 to usual care.

The trial will allow us to determine whether the MCI has an impact on BP and cardiovascular risk factors over a 2-year follow-up period and inform recommendations for health planners in scaling up these strategies for the benefit of society at large. We will also assess the effectiveness of MCI relative to usual care in terms of incremental cost per projected disability-adjusted life years (DALYs) averted and quality-adjusted life years (QALYs) saved, from the societal, payer and participant perspectives. This paper provides an analysis plan for the primary effectiveness outcome.

### Study objective

#### Primary effectiveness objective

To determine whether the effectiveness of the structured multicomponent program described above in 1–4 is better than usual care in improving the primary outcome of SBP and the secondary outcome of cardiovascular risk factors compared to usual care in the polyclinics in Singapore.

#### Hypothesis

The structured, multicomponent primary care program is better than the usual care for improving SBP among hypertensive individuals with uncontrolled hypertension.

## Study methods

### Study design

The study is planned as a cluster randomized controlled trial conducted among SingHealth Polyclinics (eight out of nine) randomized either to intervention or usual care in a 1:1 ratio.

### Randomization

The computer generated randomization will assign 4 clinics to intervention and 4 to usual care.

### Sample size and power

As suggested by epidemiological studies, including our own trials, ICCs for change in SBP in clusters of the size proposed are likely to be near 0.01 [6, 7]. Assuming a within-polyclinic ICC of 0.01 for 2-year change from baseline in SBP, a sample size of *n* = 100 patients at each of the 8 polyclinics (4 MCI, 4 usual care) achieves 80% power at *α* = 0.05 to detect a (Cohen) effect size of 0.28 between the MCI and usual care arms. An equivalent statement is that *n* = 100 patients per polyclinic achieves 80% power to reject the null hypothesis H_0_: ΔSPB_MCI_ − ΔSPB_UC_ = 0 in favor of H_1_: ΔSPB_MCI_ − ΔSPB_UC_ = δ. An effect size of *δ*/*σ* = 0.28 for a target difference of *δ* = 5 corresponds to a standard deviation of *σ* = 17.8, which is the RMSE observed in the COBRA study [8]. Allowing for 20% loss to follow-up, 125 subjects will be recruited at each polyclinic cluster. Power and Sample Size (PASS) software (PASS^14^, NCSS, Kaysville, UT, USA) was used to do the calculations.

The sample size calculations described above, performed at the time the study was being planned, were based on change from baseline in SBP as the primary outcome, consistent with similar previously conducted studies [8–10]. However, given the recently published evidence brought to our attention by a reviewer on analyses of similarly designed studies where the number of clusters is small, we concluded that repeated measures analysis on the primary outcome is likely to offer greater precision and is therefore preferable to the change in scores [11, 12]. We have therefore revised the primary outcome to 24 month SBP.

In addition, because the sample size calculation was based on change in SBP, and owing to the relatively small number of clusters, our study could be underpowered for rejecting the null hypothesis for the primary aim. In that case, failure to show a significant difference between the two groups in the primary outcome would present the possibility of an inclusive study.

### Framework

Our study is a superiority study, as the hypothesis is that MCI efficacy will be superior to usual care in improving SBP.

### Statistical interim analyses and stopping guidance

There are no plans to perform any interim analyses for assessment of primary or secondary outcomes during the study. However, analyses on unblinded data required for clinical trial monitoring activities will be performed as explained in the “Interim analyses” section.

### Timing of final analysis

All outcomes will be collectively analyzed after the final follow-up is completed at 2 years on all patients.

### Timing of outcomes assessments

Study assessments are summarized in Table 1. In the prescreening and screening assessments, individuals identified with uncontrolled high blood pressure (SBP ≥ 140 or DBP ≥ 90 mmHg) are invited to be screened for eligibility by polyclinic physicians. Successfully screened participants completing the baseline assessment will be enrolled in the study. Based on medical record information, individuals falling under the exclusion criteria will be considered screen failures and not enrolled.

**Table 1:** Scheduled assessments during study

Schedule	Baseline	Safety monitoring visit^a^	4-monthly telephone follow-up	1-year outcomes assessment (in clinic)	2-year outcomes assessment (in clinic)
Eligibility assessment	✓				
Informed consent	✓				
Blood pressure	✓			✓	✓
Anthropometric measures	✓			✓	✓
Socio-demographic information	✓				
Personal medical history	✓				
Current medication	✓^b^			✓^b^	✓^b^
Family medical history	✓				
Tobacco smoking	✓		✓	✓	✓
Physical activity	✓		✓	✓	✓
Dietary questionnaire	✓			✓	✓
EQ-5D-5L health questionnaire	✓			✓	✓
Direct and indirect healthcare cost	✓			✓	✓
10-year CVD risk score	✓			✓	✓
Home blood pressure monitoring			✓	✓	✓
Traditional medicine use	✓		✓	✓	✓
Polyclinic visit or hospitalization since last follow-up			✓	✓	✓
Panel lab tests - Serum sodium, potassium, chloride, creatinine, fasting blood glucose, total cholesterol. High-density lipoprotein cholesterol, low-density lipoprotein cholesterol, triglycerides, alanine aminotransferase, urine strip test (blood, protein), glycated hemoglobin (if patient has diabetes)	✓			✓	✓
Urine spot tests - Albumin, sodium, creatinine	✓				✓
Safety monitoring laboratory tests - Renal (serum sodium, potassium, chloride, creatinine) - Creatine kinase - Alanine aminotransferase		✓			
Adverse event reports			✓	✓	✓

^a^Safety monitoring visit-scheduled for subjects 4 weeks after initiation of ACEI/ARB antihypertensive medication or statin

^b^The medications prescribed by the treating physician for hypertension, diabetes, and cholesterol during the visit prior to the baseline visit or in person follow-up clinic visit are recorded as current medications at respective assessment

All participants will have in-person, scheduled study visits at baseline and 1- and 2-year follow-ups at the clinic with a clinical research coordinator (CRC). The acceptable window for 1- and 2-year follow-ups will be ± 6 weeks. Participants initiated on ACEi/ARB medications at MCI clinics will have an additional visit for laboratory safety monitoring tests at approximately 4 weeks after treatment initiation. CRCs will perform telephone follow-up assessments with all participants every 4 months over the 2-year study period, in addition to the in-person 1- and 2-year outcome assessments. Additional details regarding study assessment forms, questionnaires, and checklists are available in the published protocol [5].

## Statistical principles

### Confidence intervals and *P* values

Continuous variables will be summarized using conventional descriptive statistics, i.e., sample size, mean, standard deviation, median, 1st quartile, 3rd quartile, minimum, and maximum. Categorical variables will be summarized using frequencies and percentages. Percentages will typically be rounded to the one decimal place and therefore may not always sum to 100. All *p* values will be two-sided. A *p* value ≤ 0.05 will be considered statistically significant. Confidence intervals (CIs) will be calculated at the 95% level.

All subjects with significant protocol violations/deviations (see criteria below in the “Definition of protocol deviations for the trial” section) which could affect effectiveness will be identified during a blinded data review prior to data base lock.

The list of significant protocol deviation criteria will be extended as appropriate.

### Adherence and protocol deviations

#### Adherence to the intervention

MCI exposure is evaluated using the SPC prescription rate for the eligible population, delivery of motivational conversation, the telephone-based follow-up rate, and SPC subsidies. Table 2 defines these fidelity measures. The fidelity measures (95% CIs) will be estimated for the MCI arm. Exposure and adherence to anti-hypertensive medications will be summarized as secondary outcomes. Adherence will be defined as the proportion of the time that patients followed their antihypertensive medication schedules as prescribed by their physicians. Adherence will be estimated by calculating the proportion of days on which a patient had pills available during the follow-up period separated into 120-day intervals to be consistent with the follow-up visits for outcomes assessment [13, 14].

**Table 2:** Intervention component delivery (fidelity)—overall and by polyclinic

Fidelity measure	Overall	Intervention clinic 1	Intervention clinic 2	Intervention clinic 3	Intervention clinic 4
N enrolled in intervention group					
N identified as high CVD risk /N recruited in intervention group					
N eligible for MCI^a^					
**MCI-1** N of high risk subjects received motivational conversation /N Eligible for MCI					
**MCI-2** N prescribed SPC^b^ anti-hypertensive medication /N eligible for MCI					
**MCI-3** N provided SPC subsidy /N prescribed SPC antihypertensive medication					
**MCI-4** Telephone follow-up completed /N recruited in intervention					

^a^*MCI* multi component intervention

^b^*SPC* single pill combination

#### Definition of protocol deviations for the trial

Protocol deviations in the study are explained in detail below in the “Per-Protocol population” section.

### Analysis populations

#### Intention-to-treat (ITT) population

A descriptive summary of the ITT population will be provided, including significant protocol deviations by study arm. All enrolled subjects will be included in the ITT analysis. In the analysis of the ITT population, subjects will be analyzed according to study group assignment, i.e., subjects randomized to MCI clusters will be included in the MCI study group even if they did not receive the MCI. Similarly, subjects randomized to usual care clusters will be included in the usual care arm even if they are exposed to the MCI.

The ITT population data will be used in the primary analyses involving effectiveness endpoints and in the analyses of demographic characteristics, subject and family medical history, current medications, subject disposition and protocol deviations.

#### Treated (MCI) population

In the MCI arm, the treated population will include all enrolled subjects who received at least one of the components of the multicomponent intervention. In the usual care arm, the treated population will include all enrolled subjects. Participants from a MCI cluster considered not to have been “treated” will be analyzed with the usual care arm. In determining if a patient has not been “treated,” all of the following apply: received no care by a trained physician *and* did not attend a motivational conversation *and* did not receive a telephone-based follow-up from a trained nurse *and* received no subsidy on SPC antihypertensive medication as a part of the MCI intervention.

The safety analysis will be performed on the treated population data. Data from the treated population will also be used in the analysis of study intervention exposure and in sensitivity analyses of effectiveness endpoints.

#### Complete follow-up population

The complete follow-up population will include all enrolled subjects who completed the 2-year follow-up period. Data from the complete follow-up population will be used in sensitivity analyses of effectiveness endpoints.

#### Per-protocol population

The per-protocol population will include all enrolled subjects with no significant protocol deviations.

For the ITT population analysis, significant protocol violation/deviations will be identified. The latter include those which could potentially impact on the primary effectiveness measures, or present a safety risk to the participants, and/or those that are of ethical concerns.

The general significant protocol violation/deviation criteria are listed below.

- Eligibility deviations

1. Incorrect hypertensive diagnosis: Participant did not have uncontrolled BP at baseline (systolic BP ≥ 140 mmHg and/or diastolic BP ≥ 90 mmHg)
2. Physician diagnosis of hypertension not confirmed by clinic records
3. Under age: age < 40 years
4. Did not visit the polyclinic at least twice during the 1 year prior to randomization

- On-study deviations

1. Pregnancy
2. Any major medical systemic illness that precludes continuation such as terminal cancer, advanced liver disease, end-stage kidney disease.
3. Simultaneously receiving treatment at a clinic other than the study polyclinic.
4. Consent withdrawal

Data from the per-protocol population will be used in sensitivity analyses of effectiveness endpoints.

## Trial population

### Screening data

The number of subjects prescreened, proportion eligible out of number prescreened, proportion consented out of prescreened eligible, physician screen failures, and subjects who completed baseline interviews will be reported to describe the representativeness of the trial sample.

### Eligibility

#### Inclusion criteria

Study participants will be Singapore citizens or permanent residents age 40 years or older who visited the recruiting polyclinic at least twice during the 12 months prior to recruitment with two or more prior diagnoses of hypertension (SBP ≥ 140 or DBP ≥ 90 mmHg) or physician diagnosed hypertension or were on antihypertensive medications but blood pressure remained uncontrolled.

#### Exclusion criteria

Patients excluded will be those pregnant or breastfeeding, or with clinically unstable heart failure, advanced kidney disease, known liver disease, major debilitating disease, active systemic illness during the 4 months prior to recruitment, or mental illness that would invalidate informed consent.

### Recruitment

All potential participants who are eligible in pre-screening will be recruited into the study with prospective informed consent. At the time of consent, they are informed of the study details such as background, intervention components, inclusion/exclusion criteria, and follow-ups. Participants receiving usual care are informed that, as they are being cared for at a usual care clinic, they will continue their medications/treatment as per standard care, with regular outcome assessments over the study duration of 2 years, including 4 monthly telephone follow-ups and a yearly polyclinic evaluation. Standard care means that their condition will be properly managed by doctors following existing treatment guidelines and processes. In addition, they are appraised regarding their follow-up schedule, lab tests, and other information that will be requested of them. Participants in both intervention and usual care arms were recruited into the trial after obtaining written informed consent.

A CONSORT flow chart will be used to summarize the number of participants enrolled in the study at prescreening, reasons for prescreening failure, number of participants giving informed consent, physician screen failures, and final enrollment. Numbers completing baseline and follow-up visits and mean and SD of cluster size at each visit will also be presented. This chart will include all pre-screened subjects.

### Withdrawal/follow-up

The distribution of number of follow-up visits completed and disposition at the final follow-up visit (completed/died/lost-to-follow-up/protocol deviations/switching to other polyclinic/other) will be summarized by intervention arm and cluster for the ITT population. Descriptive statistics of time from baseline to first follow-up visit and time to final follow-up visit will be presented.

### Baseline patient characteristics

For the ITT population, distributions of the following variables will be summarized overall and by MCI arm using descriptive statistics:

- *Demographic characteristics*: age, gender, ethnicity, education level, marital status, employment status, home ownership, type of housing
- *Disease characteristics*: systolic and diastolic BP, uncontrolled BP (SBP > 140 mmHg and DBP > 90 mmHg), poorly controlled BP (SBP > 160 mmHg and DBP > 100 mmHg), high 10-year risk for cardiovascular disease [15], current anti-hypertensive medication status, subject and his/her family medical history, EQ-5D-5L assessment
- *Lifestyle characteristics*: current smoking status, physical activity [16] and dietary intake

#### Demographic and socio-economic characteristics

Age, gender, ethnicity, education level, marital status, employment status, house ownership, housing type, waist circumference, and BMI will be recorded at the baseline visit. The age of a subject will be defined as the truncated difference in years between the date of the baseline interview and the date of birth, plus 1 day, calculated as follows: age (years) = integer [(informed consent signed date/baseline interview date − date of birth + 1)/365.25].

#### Medical history of study subjects and family

A subject’s medical history will be recorded at baseline and include a pre-specified list of conditions (high blood pressure, heart disease, diabetes mellitus, and stroke), number of years with each condition, and current treatment (if any) the subject is taking for each condition.

Medical history of heart disease or stroke in the subject’s family (parents or siblings) will be recorded at baseline.

#### High risk for cardiovascular disease over 10 years modified for Singapore population

The 10-year cardiovascular disease risk will be calculated in the clinic per usual practice using the Framingham Risk Score [15] modified for the Singapore population. A CVD risk score ≥ 20% over 10 years is considered as “high risk.”

#### Concomitant medications

Information on any on-going medications at baseline and those received during the course of the study is collected by extracting data on medication prescriptions at the baseline and 1-year and 2-year in-clinic assessments and primarily from electronic records for conditions such as hypertension, diabetes, and high cholesterol. Medications prescribed on the visit prior to the follow-up visit will be recorded so that the data reflects medications possessed by the patient on the day of follow-up. Apart from this, data from electronic records on dispensed medications will be extracted electronically for all medications from baseline until the final assessment visit at 2 years. Medications will be classified according to the intended medical condition, such as high blood pressure, diabetes, high cholesterol, chronic kidney disease, stroke, and others. Frequency and percentage of subjects who have taken any such medications will be tabulated in the ITT population.

### Study intervention fidelity assessment

The multi-component intervention exposure will be evaluated using following fidelity measures:

- SPC prescription rates in patients classified per study algorithm as high CVD risk patients (see Table 2)
- Proportion of patients receiving nurses’ motivational conversation in high CVD risk patients,
- Proportion receiving telephone follow-up calls from nurses and average number of telephone follow-up calls per patient
- Proportion receiving subsidies for SPC among those prescribed SPC

Results for SPC prescription rates in high CVD risk patients, nurse motivational conversations at baseline with high CVD risk patients, follow-up telephone calls from nurses to participants in the intervention clinic, and subsidy provided for SPC will be reported as proportions.

### Medication adherence

Data on antihypertensive and anti-lipid medications will be obtained from SingHealth EMR clinic pharmacy records, clinic notes, and hypertensive individuals. Medication prescription information (medication name, dosage, and frequency) and medication dispensed information (medication name, dose, frequency, date of filled prescription, and number of days’ supply) will be collected and supplemented with clinic notes and an interviewer administered questionnaire. Discrepancies will be recorded and verified, e.g., if medications were purchased from a private clinic, then patient data will supersede pharmacy records.

Adherence will be defined as the proportion of the time that patients followed their antihypertensive medication schedules as prescribed by their physicians. Adherence will be estimated by calculating the proportion of days on which a patient had pills available during the follow-up period separated into 120-day intervals to be consistent with the follow-up visits for medication refills in the polyclinics. This will be divided by the number of days and multiplied by 100 to compute the percentage adherence. Average percent adherence to antihypertensive and lipid lowering medications will be computed as a composite score. The composite adherence percentage of patients to antihypertensive and lipid lowering medications will be compared between the intervention and the control groups. This approach has been used previously in other studies where adherence has been associated with CVD mortality as a means of establishing study validity and reliability [13, 14].

Antihypertensive percentage of time adherent will be calculated separately for hypertensive individuals prescribed antihypertensive medications.

## Analysis

### Outcomes

#### Primary outcome definition

Primary outcome: The primary endpoint is SBP at the final follow-up at 2 years post-randomization.

The original primary outcome for the trial was change in SBP from baseline to 24 months post randomization. However, as described above, with the expectation of achieving greater precision, the primary outcome has now been changed to SBP [11, 12].

#### Secondary outcomes

Secondary outcomes will involve comparisons between MCI and usual care for the following endpoints at the 1- and 2-year follow-up visits:

- SBP at 1-year follow-up (mmHg)
- DBP (mmHg)
- Blood pressure controlled to conventional goal (SBP < 140 mmHg and DBP < 90 mmHg)
- BP controlled to target per study algorithm
- BP controlled to target per study algorithm or ≥ 5 mmHg reduction in SBP
- Uncontrolled blood pressure (SBP > 140 mmHg and DBP > 90 mmHg)
- BP poorly controlled (SBP ≥ 160 mmHg or DBP ≥100 mmHg)
- Antihypertensive percentage of time adherent [13, 14]
- Serum LDL cholesterol (mmol/L)
- Hypertensive individuals with reduction in LDL cholesterol > 0.4 mmol/L (> 15 mg/dl)
- Composite outcome of death or hospital admission during 2-year follow-up
- Composite outcome of death or hospital admission due to CHD, heart failure, or stroke
- Individual outcomes of all-cause mortality, CVD death, and hospital admission due to CHD, heart failure, or stroke.
- Diabetic hypertensive individuals with a reduction of > 0.5% in glycated hemoglobin or a reduction in glycated % hemoglobin to < 7% in those with baseline glycated hemoglobin of ≥ 7%
- Albuminuria measured as urine albumin to creatinine ratio (ACR) (mg/g)
- Estimated CKD-EPI GFR (ml/min/1.73m^2^) [17]
- Cardiovascular risk score
  - Calculated by Framingham risk score (FRS) [15]
  - Calculated by INTERHEART risk score [18]
  - Calculated by Million Hearts Longitudinal ASCVD Risk Assessment Tool [19]
- Total cholesterol (mmol/L)
- Lifestyle (diet, physical activity based on self-report) and BMI (kg/m^2^) [16]
- Current smokers [20]
- Health-related quality of life (HRQoL) score assessed by EQ-5D-5L [21]
- Waist circumference [22]
- Fruits and vegetables intake (per week)
- Salt intake (g/day) [23]
- Incident diabetes (use of hypoglycemic agents or fasting blood glucose ≥ 126 mg/dL during 2-year follow-up for participants without prevalent diabetes at baseline)

#### Process outcomes

- Tobacco use, physical activity pattern, home BP monitoring, and antihypertensive medication use at 4, 8, 12, 16, and 24 months based on telephone follow-ups
- Number of antihypertensive medications and statins used at 2-year clinic follow-up
- Adherence to antihypertensive medications at 2-year clinic follow-up

### Analysis methods

#### Effectiveness analyses

##### Primary effectiveness analysis

While a deviation from our original planned outcome of change in SBP, the primary outcome for the trial will be mean SBP at 24-month follow-up.

Systolic BP is the primary outcome in our study. SBP at baseline and 12 and 24 months will be modeled at the subject level in a likelihood-based, linear mixed-effects model repeated measures (MMRM) analysis with cluster random effects for clinic, for participant and Gaussian error distribution with identity link function. Inferences will be based on the F-distribution (omnibus hypotheses) and the t-distribution (group comparisons at follow-ups), and as the number of clinics is small [24], degrees of freedom will be adjusted using the Satterthwaite approximation to mitigate inflation of the type I error.

The general analysis model will incorporate study group, visit and the group-by-visit interaction as fixed effects, and subjects and clinics as random effects.

Linear mixed-effects analysis model with repeated measurements:

$$ {\mathrm{SBP}}_{ijkl}=\mu +{\mathrm{Grp}}_i+{\gamma}_{(i)l}+{\mathrm{Sub}}_{(il)j}+{\mathrm{Time}}_k+{\mathrm{GT}}_{ik}+{\varepsilon}_{ijkl} $$

$$ \left(i=1,2;l=1\  to\ 4;j=1\  to\ {n}_{(il)j};k=0,1,2\right). $$

where

SBP_*ijkl*_= Systolic blood pressure

Grp_*i*_ = fixed effect of the *i*th treatment group (MCI, Usual Care)

*γ*_(*i*)*l*_ = random effect of cluster (clinic) *l* within the *i*th treatment group, where ${\gamma}_{(i)l}\sim N\left(0,{\sigma}_{\gamma}^2\right)$

Sub_(*il*)*j*_ = random effect of the *j*th subject within the *l*th clinic of the *i*th treatment group, where ${\mathrm{Sub}}_{(il)j}\sim N\left(0,{\sigma}_{\mathrm{Sub}}^2\right)$

Time_*k*_ = fixed effect of the *k*th follow-up time (baseline, 12 and 24 months)

GT_*ik*_ = treatment group × follow-up time interaction.

*ε*_*ijkl*_ = random error, where ${\varepsilon}_{ijkl}\sim N\left(0,{\sigma}_{\varepsilon}^2\right)$

The normal distribution using the identity link function will initially be employed. Our sample size is small and unlikely to yield adequate estimates of between-period ICC. Hence, we are proposing a conventional analysis using an unstructured variance-covariance matrix which accounts for a potentially varying correlation structure among subjects over follow-up times. In the event of non-convergence, other reasonable variance-covariance structures offered as options by SAS PROC GLIMMIX will be assessed and a reasonable alternative selected [25]. The rationale for selection of the variance-covariance structure will be predicated on the combined basis of model convergence, parsimony in number of parameters estimated, and the Akaike or Bayesian Information Criteria. Normality of residuals will be assessed graphically using histograms and Q-Q plots. In the event that the assumption of normally distributed errors is untenable, approximate normality of residuals will be achieved by means of transformations or alternative distributions and link functions will be used.

The general analysis model will be unconditional longitudinal data analysis (LDA) model enabling tests on study group and follow-up time main effects, group × follow-up time interaction, and group comparisons at each follow-up time. As noted by Coffman CJ et al. [11], the general test used for treatment difference over time in an unconditional LDA is equivalent to a change score type of analysis; comparing change from baseline to follow-up between randomized groups.

Baseline SBP will be included as one of the repeated outcomes measures. 95% confidence intervals will be calculated on the estimated differences [11, 12]. Based on considerations raised in papers by Hooper et al. [12] and Coffman et al. [11] using baseline as an outcome vector in an unconditional LDA is a robust analysis strategy that make no assumptions regarding equality of baseline values, which we believe is more relevant when the clusters are few in number [11, 12].

##### Adjustment for covariates

Potential covariates

Testing and adjustment for baseline covariates is generally not advocated for randomized studies. Although there are conflicting opinions regarding testing and adjusting for baseline covariates in cluster randomized studies [12, 26], we opted not to adjust for covariates in the primary effectiveness analysis. Adjustment for confounders will be done in sensitivity analyses (the “Sensitivity analysis 2” section), and baseline characteristics for the following variables will be compared between treatment groups: age (in years), gender (male, female), ethnicity (Chinese, Malay, Indian, Other), education level, employment status, house ownership, housing type, marital status, BMI/obesity status, waist circumference [27], diabetes (yes, no) (physician diagnosed or FBS > 7 mmol/L or HbA1c > 6.5% at baseline), history of cardiovascular disease (heart disease, stroke), anti-hypertensive medication use, current smoking status, physical activity score, dietary habits (whether dining at Hawker center), dietary quality (poor, good), salt intake based on urinary sodium, creatinine measurements [23], cholesterol levels, and kidney function based on eGFR and UACR.

Potential effect modifiers

Potential effect modifiers are factors that may influence the treatment effect on the outcome. For the purpose of study, we will consider certain baseline characteristics (e.g., age, gender) as potential effect modifiers. We will explore model interaction effects of potential effect modifiers with treatment effect on the primary outcome. The following variables recorded at 12 and 24 months may be considered as potential effect modifiers in the supportive analyses: BMI, waist circumference, adherence to anti-hypertensive medication, physical activity level, poorly controlled BP (systolic BP ≥ 160 mmHg or diastolic BP ≥ 100 mmHg), high CVD risk group (≥ 20% risk at 10 years on FRS, diabetes or CKD), FRS > 20% for CVD, diabetes (physician diagnosed or fasting blood sugar > 7 mmol/L or HbA1c > 6.5% at baseline), presence of CKD (eGFR < 60 ml/min/1.73m^2^ or UACR > 30 mg/mmol), smokers, and age > 65 years.

Primary analysis

MMRM model analyses will be performed on SBP for the ITT population adjusting degrees of freedom using the Satterthwaite approach; study groups will be compared using contrasts with primary interest on the 2-year follow-up means.

Additional ad hoc sensitivity analyses may be performed on the treated, per-protocol, and complete follow-up populations.

In addition, sensitivity analysis may be performed on the trial population followed up before COVID-19 national lockdown regulations were implemented on April 7, 2020, in Singapore.

##### Sensitivity analysis

Sensitivity analysis 1

MMRM model analyses will be performed on the ITT population for SBP adjusting degrees of freedom using the within-between approach; study groups will be compared using a contrast on the 2-year follow-up means.

Secondary analysis will use the same approaches on and the treated, per-protocol, and complete follow-up populations.

Sensitivity analysis 2

MMRM model analyses including and/or excluding the Time_*k*_ and GT_*ik*_ terms may be performed on the ITT population for SBP change from baseline in which potential confounders are introduced one at a time or in selected combinations.

Sensitivity analysis 3

Variance-weighted clinic-level regression on SBP change from baseline using the ITT population [24].

Additional ad hoc sensitivity analyses may be performed using the treated and per-protocol populations with adjustment for confounders.

Sensitivity analysis 4

MMRM model analyses will be performed on the ITT population for SBP adjusting degrees of freedom using the within-between approach; study groups will be compared using a contrast on the 2-year follow-up means.

Sensitivity analysis 5

Change in systolic BP at 2 years from baseline at subject level will be analyzed on the ITT population in a likelihood-based, MMRM analysis with Gaussian error distribution and identity link function with cluster random effects for clinic. As the number of clusters is small [8], degrees of freedom will be adjusted using the Satterthwaite approximation to control inflation of the type I error.

#### Secondary effectiveness analysis

Secondary endpoints and outcomes will be analyzed similarly to the primary effectiveness endpoints and outcomes as explained above, i.e., modeling the outcomes at the subject level in a likelihood-based, linear MMRM analysis with baseline measurement as a repeated measure. Continuous secondary endpoints will be analyzed similarly to the primary endpoint as described in the previous section. Non-continuous secondary endpoints will be analyzed using the MMRM structure but generalized to accommodate categorical (logit) or count (e.g., Poisson, negative binomial) endpoints by incorporating appropriate error distributions and link functions.

#### Exploratory effectiveness analysis

MMRM model analyses similar to that described above will be performed, but with the model including additional fixed effect terms for current use of anti-hypertensive medication at baseline (yes/no) and its interactions with intervention group, the purpose being to determine whether the intervention effect differs by anti-hypertensive medication use status at baseline.

Similar analysis will be performed for gender and ethnicity, poorly controlled BP status at baseline, and possibly other sub-groups among those listed below. If the interaction effects are found to be clinically meaningful, additional exploratory and sub-group analyses in the ITT population will be performed to evaluate intervention effects on the above-mentioned endpoints. Table 3 gives definitions of the above endpoints at a particular visit.

**Table 3:** Outcome/endpoint definitions for effectiveness objectives

Outcome/endpoint	Measurement and definition
Systolic and diastolic blood pressure (SBP/DBP)	Measured using a calibrated automated device (Omron HEM- 7130 Blood Pressure Monitor) with the individual in the sitting position. Three readings taken at least 3 min apart. Mean of last two readings is used as the final measurement
SBP at 1-year (mmHg)	SBP is measured at the baseline, 1-year, and 2-year clinic visits by the clinical research coordinator. At each visit, three readings are taken at least 3 min apart following a standardized protocol. The mean of the last two readings will be used as the final SBP measurement for the visit. The SBP measurements at 1-year visit will be analyzed.
DBP (mmHg)	DBP is measured at the baseline, 1-year, and 2-year clinic visits by the clinical research coordinator. At each visit, three readings are taken at least 3 min apart following a standardized protocol. The mean of the last two readings will be used as the final DBP measurement for the visit.
Blood pressure controlled to conventional goal	SBP (mean of last 2 of 3 readings) is < 140 mmHg and DBP (mean of last 2 of 3 readings) is < 90 mmHg.
Blood pressure controlled to target per study algorithm	SBP (mean of the last 2 of 3 readings) is < 140 mmHg and DBP (mean of the last 2 of 3 readings) is < 90 mmHg, low to medium risk of CVD (Framingham risk score at < 20% risk of developing a CVD event during the next 10 years, no diabetes, no pre-existing CVD, no target organ damage like LVH, retinopathy, proteinuria ≥ 300 mg/day or clinical CKD [estimated GFR < 60 ml/min/1.73 m2 or UACR indicating 24 h albumin excretion of 300 mg/day or higher]), or < 130 mmHg systolic and < 80 mmHg diastolic if high risk of CVD (Framingham risk score at ≥ 20% risk of developing a CVD event during the next 10 years, or with diabetes or with pre-existing CVD, or with target organ damage like LVH, retinopathy, proteinuria ≥ 300 mg/day or clinical CKD)
Blood pressure controlled to target as per study algorithm or ≥ 5 mmHg reduction in SBP	BP controlled to target as explained above or reduction of SBP (mean of the last 2 of 3 readings) > 5 mmHg from baseline to the follow-up
Uncontrolled blood pressure	SBP (mean of last 2 of 3 readings) is ≥ 140 mmHg or DBP (mean of last 2 of 3 readings) ≥ 90 mmHg
Poorly controlled blood pressure	SBP (mean of last 2 of 3 readings) is ≥ 160 mmHg or DBP (mean of last 2 of 3 readings) ≥ 100 mmHg
Antihypertensive percentage of time adherent	Antihypertensive percent adherence is defined as the percentage of time hypertensive individuals followed their prescribed antihypertensive medication schedules. Percentage antihypertensive adherence will be obtained by dividing the number of days during follow-up for which a patient had pills available (based on 120-day intervals) by the total number of follow-up days. 120-day intervals are consistent with time between medication refills in the polyclinics [13, 14].
Serum LDL cholesterol (mmol/L)	Serum LDL cholesterol is collected as part of routine hypertension/diabetes lab panel in the polyclinics. Measurements will be recorded by CRC at baseline, year 1, year 2 follow-up visits, measured as total cholesterol, high-density lipoprotein cholesterol, low-density lipoprotein cholesterol, and triglycerides.
Hypertensive individuals with reduction in LDL cholesterol > 0.4 mmol/L (> 15 mg/dl)	Serum LDL cholesterol is collected as part of routine hypertension/diabetes lab panel in the polyclinics. Measurements will be recorded by CRC at baseline, year 1, and year 2 follow-up visits. The proportion of individuals experiencing a > 0.4 mmol/L decrease in LDL cholesterol at 1-year and 2-years follow-up
Composite outcome of death or hospital admission during the 2-year follow-up period	Information on the events of death or hospital admissions will be captured on study adverse event reporting forms and will be used to derive the composite outcome of death or hospitalizations caused by any condition.
Composite outcome of death or hospital admission due to CHD, heart failure, or stroke.	Information on the events of death or hospital admission due to CHD, heart failure, or stroke will be captured on study adverse event reporting forms and will be used to derive the composite outcome of death or cause-specific hospitalizations due to CHD, heart failure, or stroke.
Individual outcomes of all-cause mortality, CVD deaths, and hospital admission due to CHD, heart failure, or stroke.	Information on the events of death or hospital admission due to CHD, heart failure, or stroke will be captured on study adverse event reporting forms and will be used to derive the individual outcomes of all-cause mortality or cause-specific hospitalizations of CHD, heart failure, or stroke.
Diabetic hypertensive individuals with a reduction of ≥ 0.5% in glycated hemoglobin or a reduction of glycated % hemoglobin to < 7% in those with baseline glycated hemoglobin of ≥ 7%	Glycated hemoglobin measurements are performed in patients with diabetes as part of the routine diabetes lab panel in the polyclinics. These measurements will be recorded for participants with diabetes by the CRC at baseline, year 1, and year 2 follow-ups.
Albuminuria (measured as ACR) (mg/g)	Spot urinary albumin and creatinine tests are performed as study-specific tests for this study and will be used to calculate the urinary albumin to creatinine ratio (ACR) at the baseline and year 2 follow-up visits.
Estimated CKD-EPI GFR (ml/min/1.73m^2^)	Serum creatinine measurements are collected as part of the routine hypertension/diabetes lab panel in the polyclinics. These measurements will be recorded by the CRC at baseline, year 1, and year 2 follow-up visits and used to estimate eGFR using the CKD-EPI equation [17]
Cardiovascular risk score	The Framingham risk score with appropriate adjustments for the Singaporean population is calculated as part of routine practice in the polyclinics and will be collected by the CRC at baseline, 1-year, and 2-year follow-up visits. The original Framingham risk score without adjusting for Singaporean population [15] will be computed too. The INTERHEART “Cholesterol” modifiable risk score provides a comprehensive numeric assessment of risk factors for cardiovascular events. The score is the sum of points for questions corresponding to categories of these risk factors. In addition, Million Hearts Longitudinal ASCVD Risk Assessment Tool will be used to compute change in CVD risk [19].
Total cholesterol levels (mmol/L)	Measured in terms of total cholesterol, high-density lipoprotein cholesterol, low-density lipoprotein cholesterol, and triglycerides. (Chemistry analyzer: Roche Hitachi 912; Reagent: Roche reagents).
Lifestyle—diet	Diet: A dietary questionnaire is administered at baseline and the 1-year and 2-year follow-up visits to collection information on dietary habits. Frequency of intake per week will be considered as an indicator for each type of dietary intake.
Lifestyle—physical activity based on self-report International Physical Activity Questionnaire [16]	Physical activity: The International Physical Activity Questionnaire [16] is administered at baseline, 1-year, and 2-year follow-up visits to assess subjects’ physical activity level in their everyday life. It asks questions about the time spent being physically active in the last 7 days. It has three domains—vigorous, moderate, walking and sitting, with each having two questions. Total physical activity score (MET-min/week) and activity classification (inactive, minimally active, and highly active) are derived according to the IPAQ scoring guideline [15]. Domain score for vigorous, moderate, and walking are calculated using the following formulas. They represent volume of activity computed by weighting each type of activity by its energy requirements defined in METS (METs are multiples of the resting metabolic rate) to yield a score in MET-minutes. Vigorous MET-minutes/week = 8.0 × vigorous-intensity activity minutes × vigorous-intensity days Moderate MET-minutes/week = 4.0 × moderate-intensity activity minutes × moderate days Walking MET-minutes/week = 3.3 × walking minutes × walking days A combined total physical activity MET-min/week is computed as the sum of vigorous + moderate + walking MET-minutes/week scores. (The IPAQ Sitting question is an additional indicator variable and is not included as part of any summary score of physical activity.) Activity classifications are defined as follows: Inactive— • No activity reported, **OR** • Some activity reported but insufficient for moderately active or highly active categories Moderately active—any of the following 3 criteria: • 3 or more days of vigorous activity of at least 20 min per day, **OR** • 5 or more days of moderate-intensity activity or walking of at least 30 min per day, **OR** • 5 or more days of any combination of walking, moderate-intensity, or vigorous-intensity activities achieving a minimum of at least 600 MET-min/week. Highly active—any one of the following 2 criteria: • Vigorously intense activity on at least 3 days and accumulating at least 1500 MET-minutes/week, **OR** • 7 or more days of any combination of walking, moderate-intensity or vigorous intensity activities achieving a minimum of at least 3000 MET-minutes/week
Body mass index (BMI) (kg/m^2^)	BMI is calculated as weight (kg) divided by height^2^ (meters). Height is measured using standardized Portable Stadiometer available in polyclinics in cm with graduation of 1 mm. Weight is measured using standardized Tanita Digital Weight Scale (Tanita HS 302).
Current smokers*	Subjects smoking tobacco on a daily basis, including cigarette, pipes, cigars, cheroots, cigarillos, and water pipe smoking sessions are considered as current smokers. Modified from Global Adult Tobacco Survey Collaborative Group [20].
HRQoL/QALY	HRQoL is measured using the EQ-5D-5L questionnaire. It measures health status on the day of assessment and has five dimensions: mobility, self-care, usual activities, pain/discomfort, and anxiety/depression. Each has one question with five levels of severity. In addition, it has a visual analog scale [15] measuring health on a scale of 0 (worst health) to 100 (best health). The EQ-5D index summarizing health profile of the subjects will be calculated using the EQ-5D-5L valuation set for England [21]. QALY will be estimated using the EQ-5D scale which provides a standard metric for comparing health effects of varied interventions across diverse diseases and conditions.
Waist circumference	Measured as per the WHO STEPS protocol [22]. The measurement of waist circumference is made at the approximate midpoint between the lower margin of the last palpable rib and the top of the iliac crest.
Fruits and vegetables intake (per week)	A dietary questionnaire is administered to collect information on dietary habits related to fruits and vegetables intake. At least one intake per week will be considered an indicator for each type of dietary intake.
Salt intake (g/day)	Measured in terms of urine spot sodium-to-creatinine ratio and 24-h urine sodium estimation by Kawasaki formula [23].
Incident diabetes	The use of hypoglycemic agents or fasting blood glucose ≥ 126 mg/dL at any time during the 2-year follow-up period for all participants without prevalent diabetes at enrolment.

#### Mediation analysis

If the intervention is effective, an exploratory medication analysis will be conducted with the objective to estimate the extent to which changes in lifestyle factors (body mass index, waist circumference, diet, physical activity) and antihypertensive pharmacologic therapy (use of single pill combination antihypertensive, the number of antihypertensive medications use, antihypertensive medication dose) mediate the effect of SingHypertension MCI versus usual care on reducing BP levels.

##### Methods

We will construct multiple mediator models to understand the complex relationships among potential mediators that intervention is expected to influence including (BMI, diet, physical activity,) and pharmacologic therapy (the number, type, dose of antihypertensive and SGLT2i, and statins) for its effect on the primary outcome of change in systolic BP. Age, ethnicity, SES, CVD risk score, and co-morbidities will be considered as potential confounders. Serial multiple mediator models will also be explored, if indicated. The PROCESS macro in SAS will be used to perform the mediation analysis [28–30].

#### Safety analysis

##### Adverse events, serious adverse events, and deaths

All on-study adverse events reported on AE reporting forms will be summarized by study group. The percentage and frequency of subjects who report AEs will be tabulated for AEs, SAEs, study-related AEs, and AEs related to anti-hypertensive and anti-lipid medications. All deaths, together with the cause of death, will be listed by subject.

#### Interim analyses

Planned interim analyses are to occur every 6 to 9 months from the start of the study. Interim analyses will summarize participant baseline characteristics and safety data by study group and for pooled data from both study groups. However, no evaluation of primary outcome (intervention effect) will be performed in the interim analyses. Access to the unblinded interim data and results will be limited to the Data and Safety Monitoring Board (DSMB) members and statisticians performing the analysis. An independent DSMB will review the data as per the DSMB Charter. The results of the pooled analysis will be presented to the study team. Following the unblinded review of the safety data, the DSMB will make a recommendation to either (1) allow the study to continue according to protocol, (2) allow the study to continue with modifications to the protocol, or (3) discontinue the study in accordance with good medical practice.

### Missing data

#### Handling of missing data in the effectiveness analyses

Data for primary and secondary outcomes are collected at the 12- and 24-month in-clinic follow-up visits. It is anticipated that the primary cause of missing data will be due to subjects not completing the study and/or missing follow-up visits. A subject may miss one or both in-clinic follow-up visits owing to study withdrawal, adverse events, or death. The MMRM model employed in the primary analysis is valid under the missing at random (MAR) assumption [31]. This model assumes that once the appropriate observed data and covariates are accounted for in the model, the missingness is independent of the unobserved outcome values. We anticipate very few deaths over the course of the study period and that deaths will be MAR and noninformative, i.e., unrelated to study interventions. If warranted, sensitivity analyses investigating and comparing missingness patterns may be performed to evaluate the robustness of the MAR assumption relative to the ITT population.

#### Missing data and exclusions

All observed data will be included in the ITT analysis with the exception of data collected outside the acceptable assessment window (± 12 weeks) for each a time point. This window was extended to ± 12 weeks from ± 6 weeks, to account for the impact of the COVID-19 pandemic on follow-ups.

The primary analysis will be performed on the basis of the intention-to-treat principle (ITT) using a likelihood-based, general linear mixed-effects model for repeated measures (MMRM) based on a participant-level analysis. The MMRM maximum likelihood estimation approach for continuous outcomes naturally accounts for missing at random (MAR) data patterns. In addition, baseline covariates predictive of missing outcomes will be added to the model in a sensitivity analysis. Because this study used a small number of clinics [8], analyses incorporating adjusted degrees of freedom using the Satterthwaite approach [24] will be performed as a mitigating measure against type I error inflation. It is anticipated that the primary cause of missing data will be patients dropping out prior to the 2-year follow-up. The anticipated pattern is that once a patient misses a visit data will be missing for subsequent visits. In order to evaluate potential biases in the ITT analysis results resulting from the missing data, sensitivity analyses based on per-protocol and treated populations will be conducted and results compared to the ITT analysis results. Consistency of inferences and conclusions among results obtained from these analyses will form the basis for study inferences regarding MCI intervention effectiveness and efficacy, safety, generalizability, and validity. In the event that missingness exceeds 20%, a multiple imputation sensitivity analysis on the primary outcome may be considered.

#### Conventions and imputation of missing dates

When converting a number of days to other units, the following conversion factors will be used: 1 year = 365.25 days; 1 month = 30.4375 days. The following conventions will be used for imputing partial dates (unless otherwise specified):

- If only the day of the month is missing, the 15th of the month will be used to replace the missing day.
- If both the day and the month are missing, “July 1” will be used to replace the missing information.
- If a date is completely missing or the year is missing, it will be considered as missing.

For date of death, the following conventions will be used for imputing partial dates:

- If only the day of the month is missing, the 1st of the month will be used to replace the missing day.
- If both the day and the month are missing, “January 1” will be used to replace the missing information.

The imputed death date will be compared with the last known alive date (date of censoring for survival). The maximum of the (imputed death date, date of censoring for survival + 1) will be considered as the date of death.

An AE missing a severity level will be deemed “severe,” and an AE with a missing relationship to study drug will be deemed “definitely related.”

A Kaplan-Meier plot will be used to summarize time from enrolment to study discontinuation by study arm for the ITT population. Subjects completing the study without early withdrawal will be censored on the last visit date. Median time to study discontinuation will be calculated for each study group. A log-rank test will be performed to assess comparability of time to study discontinuation between the intervention groups.

### Harms

#### Safety objectives and endpoints

Adverse event (AE) data will be collected through one additional month beyond the 2-year follow-up. Adverse events will be classified as serious adverse events [32] and other adverse events (other AE). An event is considered a SAE if it leads to one or more of the following: death, a life threatening event, permanent disability, hospitalization, or prolongation of hospitalization (≥ 24 h). Events not meeting the definition of SAE will be classified as Other AEs. Upon occurrence of an AE, the site investigator determines category and system organ class. Adverse events are categorized based on the systems involved and are adjudicated by site PIs. Table 4 gives predefined categories and system organ classes for adverse events.

**Table 4:** Predefined event categories and system organ classes for adverse events

(a) Event categories	(b) System organ classes
Angioedema and anaphylactic reaction Peripheral edema Hypotension Coronary heart disease Heart failure Stroke or transient ischemic attack Headache Dizziness or lightheadedness Flushing Cough after initiating antihypertensive Abdominal pain Muscle pain Falls and trauma Other	Systemic reactions Cardiovascular system Nervous systems Skin and appendages Respiratory system Gastrointestinal and hepatobiliary system Musculoskeletal system Electrolyte abnormalities Other

AE data will be collected on the following pre-defined conditions: angioedema and anaphylactic reaction, peripheral edema, hypotension, coronary heart disease, heart failure, stroke or transient ischemic attack, headache, dizziness or lightheadedness, flushing, cough after initiating antihypertensive, abdominal pain, muscle pain, injuries/falls and trauma, bradycardia, and others.

The following pre-defined categories are set forth to describe the relationship of an AE to anti-hypertensive and anti-lipid medications: “definitely related,” “probably related,” “possibly related,” “unlikely to be related,” or “not related.” Events marked as “definitely related,” “probably related,” and “possibly related” will be categorized as “event related to anti-hypertensive and anti-lipid medication.” All adverse events reported with an onset date before the study consent date or 30 days after the final assessment visit (year 2 visit) will not be considered as on-study adverse events. Adverse event severity will be categorized as “mild,” “moderate,” or “severe.”

In the interest of test sensitivity for detecting differences in adverse events and safety outcomes, proportions of patients experiencing the following adverse or safety endpoints during the 2-year follow-up period will be compared between MCI and usual care study groups using a chi-squared test or Fisher’s exact test:

- AE, study-related or otherwise
- SAE, study-related or otherwise
- Study-related AE
- Study-related SAE
- Study-related AE resulting in death
- Study-related AE resulting in MCI treatment discontinuation
- AE related to anti-hypertensive or anti-lipid medications
- All-cause mortality
- All-cause mortality or hospitalization
- SAE associated with specific condition of interest (e.g., cardiovascular death, hospitalization for cardiovascular diseases due to stroke, acute coronary syndrome, elective revascularization, myocardial infarction, heart failure, and peripheral vascular disease)
- Hospitalization associated with specific condition of interest (e.g., hypotension, dizziness/lightheadedness, injury/fall, eGFR decline by > 20% in 1–30 days (AKI) or serum potassium abnormality (< 3 or > 5.5 mmol/L))
- AE associated with specific condition of interest (e.g., hypotension, dizziness/lightheadedness, injuries/falls, bradycardia, cough after initiating antihypertensive, peripheral edema, musculoskeletal pain)
- AE requiring a visit to doctor or ED associated with specific condition of interest (e.g., hypotension, dizziness/lightheadedness, injuries/falls, bradycardia, cough after initiating antihypertensive, peripheral edema, musculoskeletal pain, and associated with lab abnormalities as serum creatinine (> 20% of normal), serum sodium (< 130 or > 150 mmol/L), serum potassium (< 3 or > 5.5 mmol/L))

#### Adverse events of specific interest

Among the SAE, events of specific interest are deaths (all-cause); hospitalizations due to vascular disease, cardiovascular diseases (acute coronary syndrome, elective revascularization, myocardial infarction, peripheral vascular disease), heart failure, and stroke; and AEs related to anti-hypertensive and anti-lipid medications (hospitalizations associated with hypotension, dizziness/lightheadedness, injuries/falls, lab abnormalities (eGFR decline by > 20% in 1–30 days) or serum potassium abnormalities (< 3 or > 5.5 mmol/L)). Among the other AEs not requiring hospitalization and events related to anti-hypertensive and anti-lipid medications of interest are hypotension, dizziness/lightheadedness, injuries/falls, bradycardia, cough after initiating antihypertensive, peripheral edema, musculoskeletal pain, lab abnormalities, e.g., serum creatinine > 20% of normal, serum sodium < 130 or > 150 mmol/L, and serum potassium < 3 or > 5.5 mmol/L.

Details of conditions listed under other AEs will be sub-classified into events requiring or not requiring a doctor/A&E visit. All adverse events of specific interest will be verified from source data. Any discrepancy in AE classification will be identified and reclassified by the Chief PI.

### Statistical software

All statistical analyses will be performed using SAS software (SAS V9.4, SAS Institute, North Carolina, USA).

### Quality control of statistical analyses

All the statistical analyses and derived variables related to the primary objective will be validated by independent programming. Remaining analyses and derived variables will be validated either by independent programming or through code review. All the analyses will be carried out as per the latest version of the statistical analysis plan. While table shells and mock figures are presented here, the final tables and figures used to report findings from this study may differ. Details of validation methods and outcomes will be documented.

## Discussion

Our proposed trial is novel in its comprehensive package of up-to-date, evidence-based potentially sustainable and scalable strategies to lower BP, lipids, and cardiovascular risk among individuals with uncontrolled hypertension visiting the public-sector polyclinics in Singapore. The major components emphasize algorithm-based treatment using SPC antihypertensive medications and statins as first-line agents in all high-risk hypertensive individuals, motivational counseling for enhancing adherence, physician-supervised health workers, and structured remote telephone follow-up by nurses with a focus on cardiovascular risk reduction. Additionally, the intervention strategies are integrated into the existing health system and include measures for internal monitoring and quality control as well as robust method of evaluation to inform clinical effectiveness and cost-effectiveness.

Our trial is not powered to assess the hard outcomes of CVD morbidity and mortality, as such a study would require a much larger sample size as well as longer duration. Nevertheless, there is unequivocal epidemiological and trial evidence that a reduction in systolic BP, the primary outcome of our trial, is strongly correlated with reducing risk of CVD. Furthermore, our proposed primary and secondary outcomes are known to modify CVD risk substantially.

The major limitations of the analysis are the small number of clusters (clinics) reducing the power of study, despite including all eight polyclinics in one large administrative health network. Of note, increasing the cluster size (number of patients) has little effect on the power of a cluster RCT. Moreover, fewer clusters increase the likelihood of imbalance at randomization and possibility of confounding Furthermore, while a deviation from our original analytic plan, the primary outcome of our trial is mean SBP at 24-month instead of change in SBP from baseline. We will employ contemporary analytic approach of unconditional longitudinal data analysis that make no assumptions about baseline characteristics and includes baseline measurement as one of the repeated outcomes measures [11, 12]. This approach is likely to offer more precise estimates than originally planned change in 24 h SBP. Thus, we believe our analysis will be robust.

A major strength of our trial is the cluster design randomized at the polyclinic level which mitigates the risk of intervention contamination among clinic regions and permits an unbiased evaluation of the intervention at either the individual or clinic level.

The primary efficacy analysis will be performed under the ITT principle using a mixed-effect individual-based analysis with Satterthwaite adjustment to degrees of freedom to mitigate inflating the type I error. The efficacy estimate from this analysis will reflect the effects of protocol deviations and varying levels of intervention adherence/non-adherence. A similar per-protocol analysis will provide a more optimistic efficacy estimate. Additional clinic-level sensitivity analyses will allow assessment of the mixed model assumptions on the efficacy estimates.

As the MCI involves components such as motivational conversation for high-risk hypertensive individuals by trained nurses, and telephone-based follow-ups of all hypertensive individuals by polyclinic nurses, we expect that there will be some over- and under-enthusiastic nurses; also, variability in adherence to the study treatment algorithm by physicians means the effectiveness of the MCI may be impacted. However, as the intervention is designed to roll out for a population-wide program, variation in performance of individual nurses and physicians are of limited interest. Therefore, our primary analytic approach does not consider multilevel modeling incorporating such micro-level effects. However, the main analysis is designed to incorporate cluster or clinic level effects in the analysis.

In summary, our statistical analysis plan presents the approach for analyzing the real-world impact of the MCI in polyclinic in Singapore before any post-baseline outcomes for effectiveness have been analyzed. By reporting an a priori statistical analysis plan, we will avoid reporting bias and data-driven analysis for the primary and key secondary effectiveness outcomes in reporting our trial results.

Thus, our proposed trial is anticipated to have significant clinical practice and public health policy implications in Singapore and globally.

## Data Availability

This article describes only statistical methodologies without reporting any study data. The study data will be available on request from the primary investigator after completion of the study.

## Abbreviations

ACEi: Angiotensin-converting enzyme inhibitor
ARB: Angiotensin receptor blocker
AE: Adverse event
AKI: Acute kidney injury
BMI: Body mass index
BP: Blood pressure
CRC: Clinical research coordinator
CVD: Cardiovascular diseases
DALYs: Disability-adjusted life years
DBP: Diastolic blood pressure
DSMB: Data and Safety Monitoring Board
eGFR: Estimated glomerular filtration rate
EQ-5D-5L: EuroQol-5 Dimension-5 Level questionnaire
FBS: Fasting blood sugar
FRS: Framingham Risk Score
HRQoL: Health-related quality of life
ICC: Intraclass correlation coefficient
IPAQ: International physical activity questionnaire
LDL: Low-density lipoproteins
ITT: Intention-to-treat
MAR: Missing at random
MCI: Multicomponent intervention
MMRM: Mixed-model for repeated measures
PASS: Power and sample size
QALYs: Quality-adjusted life years
SPC: Single pill combination
SAE: Serious adverse event
SBP: Systolic blood pressure
SD: Standard deviation
UACR: Urine albumin-to-creatinine ratio
