# Correction to: Statistical analysis plan for management of hypertension and multiple risk factors to enhance cardiovascular health in Singapore: the SingHypertension pragmatic cluster randomized controlled trial

**DOI:** 10.1186/s13063-021-05097-9

**Published:** 2021-02-08

**Authors:** John C. Allen, Benjamin Halaand, Rupesh M. Shirore, Tazeen H. Jafar, for SingHypertension Study Group

**Affiliations:** 1 Centre for Quantitative Medicine, Duke-NUS Medical School, Level 6, Academia, 20 College Road, Singapore, Singapore; 2 Division of Biostatistics, Population Health Sciences, University of Utah, Salt Lake City, USA; 3 Program in Health Services & Systems Research, Duke-NUS Medical School, 8 College Road, Singapore, Singapore

**Correction to: Trials (2021) 22:66**

**https://doi.org/10.1186/s13063-020-05016-4**

Following publication of the original article [1], we were notified that a citation was inadvertently added in the abstract. The publisher apologizes for any inconvenience.

The original article has been corrected.
